# Epidemic Microclusters of Blood-Culture Proven Sepsis in Very-Low-Birth Weight Infants: Experience of the German Neonatal Network

**DOI:** 10.1371/journal.pone.0038304

**Published:** 2012-06-29

**Authors:** Christoph Härtel, Kirstin Faust, Stefan Avenarius, Bettina Bohnhorst, Michael Emeis, Corinna Gebauer, Peter Groneck, Friedhelm Heitmann, Thomas Hoehn, Mechthild Hubert, Angela Kribs, Helmut Küster, Reinhard Laux, Michael Mögel, Dirk Müller, Dirk Olbertz, Claudia Roll, Jens Siegel, Anja Stein, Matthias Vochem, Ursula Weller, Axel von der Wense, Christian Wieg, Jürgen Wintgens, Claudia Hemmelmann, Arne Simon, Egbert Herting, Wolfgang Göpel, for the German Neonatal Network (GNN)

**Affiliations:** 1 Department of Pediatrics at the University of Lübeck, Lübeck, Germany; 2 Department of Pediatrics at the University of Magdeburg, Magdeburg, Germany; 3 Department of Pediatrics at the Medical School Hanover, Hanover, Germany; 4 Department of Neonatology, Vivantes-Klinikum Neukölln, Berlin, Germany; 5 Department of Pediatrics at the University of Leipzig, Leipzig, Germany; 6 Department of Neonatology, Klinikum Leverkusen, Leverkusen, Germany; 7 Department of Neonatology, Klinikum Dortmund, Dortmund, Germany; 8 Department of Pediatrics at the University of Düsseldorf, Düsseldorf, Germany; 9 Department of Neonatology, Klinikum Siegen, Siegen, Germany; 10 Department of Pediatrics at the University of Cologne, Cologne, Germany; 11 Department of Pediatrics at the University of Göttingen, Göttingen, Germany; 12 Department of Neonatology, Asclepios Klinik Barmbek, Hamburg, Germany; 13 Department of Pediatrics at the University of Dresden, Dresden, Germany; 14 Department of Neonatology, Klinikum Kassel, Kassel, Germany; 15 Department of Neonatology, Südtstadt Klinik Rostock, Rostock, Germany; 16 Department of Neonatology, Vestische Klinik, Datteln, Germany; 17 Department of Neonatology, Krankenhaus Aud der Bult, Hanover, Germany; 18 Department of Pediatrics at the University of Essen, Essen, Germany; 19 Department of Neonatology, Olga-Hospital, Stuttgart, Germany; 20 Department of Neonatology, Klinikum Bielefeld, Bielefeld, Germany; 21 Department of Neonatology, Altonaer Kinderkrankenhaus, Hamburg, Germany; 22 Department of Neonatology, Klinikum Aschaffenburg, Aschaffenburg, Germany; 23 Department of Neonatology, Städtisches Klinikum Rheydt, Mönchengladbach, Germany; 24 Institute of Biometry and Medical Statistics at the University of Lübeck, Lübeck, Germany; 25 Department of Pediatric Hematology and Oncology at Saarland University Hospital, Homburg, Germany; Erasmus University Rotterdam, The Netherlands

## Abstract

**Introduction:**

We evaluated blood culture-proven sepsis episodes occurring in microclusters in very-low-birth-weight infants born in the German Neonatal Network (GNN) during 2009–2010.

**Methods:**

Thirty-seven centers participated in GNN; 23 centers enrolled ≥50 VLBW infants in the study period. Data quality was approved by on-site monitoring. Microclusters of sepsis were defined as occurrence of at least two blood-culture proven sepsis events in different patients of one center within 3 months with the same bacterial species. For microcluster analysis, we selected sepsis episodes with typically cross-transmitted bacteria of high clinical significance including gram-negative rods and *Enterococcus spp*.

**Results:**

In our cohort, 12/2110 (0.6%) infants were documented with an early-onset sepsis and 235 late-onset sepsis episodes (≥72 h of age) occurred in 203/2110 (9.6%) VLBW infants. In 182/235 (77.4%) late-onset sepsis episodes gram-positive bacteria were documented, while coagulase negative staphylococci were found to be the most predominant pathogens (48.5%, 95%CI: 42.01–55.01). *Candida spp*. and gram-negative bacilli caused 10/235 (4.3%, 95%CI: 1.68% –6.83%) and 43/235 (18.5%) late-onset sepsis episodes, respectively. Eleven microclusters of blood-culture proven sepsis were detected in 7 hospitals involving a total 26 infants. 16/26 cluster patients suffered from *Klebsiella spp.* sepsis. The median time interval between the first patient’s *Klebsiella spp.* sepsis and cluster cases was 14.1 days (interquartile range: 1–27 days). First patients in the cluster, their linked cases and sporadic sepsis events did not show significant differences in short term outcome parameters.

**Discussion:**

Microclusters of infection are an important phenomenon for late-onset sepsis. Most gram-negative cluster infections occur within 30 days after the first patient was diagnosed and *Klebsiella* spp. play a major role. It is essential to monitor epidemic microclusters of sepsis in surveillance networks to adapt clinical practice, inform policy and further improve quality of care.

## Introduction

Sepsis is still one of the leading causes of mortality, long-term morbidity including CNS and lung disease and prolonged stay in hospital in very low-birth-weight (VLBW) infants [1]–[3]. According to data from the NICHD network, blood-culture-proven late-onset sepsis affected 21% of VLBW infants, with many more neonates receiving empirical antibiotic treatment for presumed or possible sepsis. Late-onset sepsis incidence varies considerably among centers involving 11 to 32% of infants at risk [4]–[5], even after adjustment for known risk factors such as gestational age, outborn status, parenteral nutrition or mechanical ventilation [6]. The implication is that this variation may be associated with differences in clinical practices and NICU settings. In line with this, periodic overcrowding (high bed occupancy) and understaffing (low nurse: patient ratio) are well documented risk factors for nosocomial infections and occurrence of outbreaks [7]–[10]. Monitoring the epidemiology of late-onset sepsis including the spectrum of pathogens in blood cultures and antibiotic resistance patterns over time is essential for design and implementation of prevention [11]. Infection surveillance networks are well established in many countries including USA (NICHD; Vermont-Oxford [1], Israel [2], Australasia [12], Canada [6], England (NeonIn; [13]) and Germany (NeoKiss, [14]). Despite these efforts it is still unknown to what extent late-onset sepsis occurs in epidemic microclusters and which impact sepsis clusters may have on outcome of VLBW infants [15]. This is of particular importance for gram-negative sepsis clusters which are less prevalent but associated with increased mortality (19–36%, [1], [16]).

The German Neonatal Network (GNN) is a prospective cohort study with the focus on long term development of VLBW infants. The multicenter collaboration currently includes 46 tertiary level neonatal units in Germany and provides a platform for benchmarking practice, interventional trials and the investigation of genetic and clinical risk factors of VLBW infants. In a previous study of our group (2003–2008; 16 participating centers), we noted a total sepsis risk of 16.8% in 2995 VLBW infants and a risk of 14.8% to suffer from late-onset sepsis with center-to-center variation from 4–26%. More than 80% of late-onset sepsis episodes occurred between day 4 and day 30 of life [2]. This report describes results of the German Neonatal Network (GNN) in the study period 2009–2010. We specifically determined cases of blood-culture proven clinical sepsis with typical cross-transmitted gram-negative bacteria and *Enterococci* which occurred in timely association in the same NICU (epidemic microcluster). We also evaluated outcome of cluster-involved patients compared to single cases with sepsis.

## Methods

### Ethical Approval

Ethical approval was given for all study parts by the University of Lübeck Ethical Committee^1,24^ and by all local ethical committees at the participating study centres. Specifically: Ethical Board of the University of Magdeburg^2^, Ethical Board of the Medical School Hannover^3,17^, Ethical Board of the University of Leipzig^5^, Ethical Board of the University of Düsseldorf^8^, Ethical Board of the University of Cologne^10^, Ethical Board of the University of Göttingen^11^, Ethical Board of the University of Dresden^13^, Ethical Board of the University of Essen^18^; Ethical Board of the Medical Chamber of Berlin^4^, Ethical Board of the Medical Chamber of the North Rhine region^6,7,23^, Ethical Board of the Medical Chamber of the Westphalia-Lippe region^9,20^, Ethical Board of the Medical Chamber of Hamburg^ 12,21^, Ethical Board of the Medical Chamber of the federal state of Hessen^14^, Ethical Board of the University of Rostock^15^, Ethical Board of the University of Witten-Herdecke^16^, Ethical Board of the Medical Chamber of the federal state of Baden-Württemberg^19^, Ethical Board of the Medical Chamber of the federal state of Bavaria^22^ and Mönchengladbach^23^, Ethical Board of the Saar University^25^.

Informed written consent was given by all parents (as legal representative) on behalf of their infants.

### Study Population

We prospectively determined the primary outcome data in 2384 VLBW infants enrolled in GNN by 37 level III neonatal intensive care units during the study period January 2009 until December 2010. The inclusion criteria were: birth weight <1500 g and gestational age ≤36+6 weeks. After informed written parental consent of infants, a predefined clinical data set of 220 parameters is recorded for each patient on clinical record files. In all infants born in GNN centers but not enrolled in GNN a basic data set including the parameter “blood-culture proven sepsis” was documented.

### Definitions

The centers were asked to document clinical episodes of infection as defined by NeoKISS criteria (2 clinical signs, 1 laboratory sign, 5 days of antibiotic therapy; [14], [17]) and blood-culture proven clinical sepsis. In this report, we focused on the sepsis definition of “clinical sepsis with positive blood culture” (bloodstream infection). Late-onset sepsis was defined as blood-culture proven clinical sepsis occurring ≥72 hours of age.

Microclusters of sepsis were defined as occurrence of at least two blood-culture proven sepsis events in one center within 3 months with the same bacterial species in different patients. For microcluster analysis, we selected sepsis episodes with typical cross-transmitted bacteria of high clinical significance incl. gram-negative rods and *Enterococcus spp*.

Data quality was approved by regular on-site monitoring of participating centers by a physician trained in neonatology including cross-check of local microbiological read-outs.

### Data Entry

All data were entered in an Access database by health record administrators at the main GNN-office at the University of Lübeck. After discharge, clinical record files of the participating hospitals are sent to the study centre.

### Statistical Analysis

Data analysis was performed using the SPSS 20.0 data analysis package (Munich, Germany) and SAS 9.3. Hypotheses were evaluated with Fisher’s exact test and Mann-Whitney U test. Furthermore, the generalized estimating equations with an independent working correlation structure were used for the estimates of the proportions and the corresponding confidence intervals. A *p* value <0.05 was considered as statistically significant for single tests.

## Results

### Epidemiology of Blood-culture Proven Sepsis

3661 VLBW infants were eligible for enrolment in the GNN study period 2009/2010. Of these, 416 (11.4%) had at least one episode of blood-culture proven sepsis. 2384/3661 (65.1%) VLBW infants were recruited in 37 GNN centers and had a total rate of blood-culture proven sepsis of 10.5% (250/2384; 0.6% of infants had an early-onset sepsis, 9.9% suffered from a late-onset sepsis).

In order to analyse microclusters of sepsis, we decided to exclude all infants born in those centers who had recruited less than 50 VLBW infants in the study period, therefore 2110 VLBW infants born in 23 centers remained for further analysis. In this cohort, the incidence density for sepsis was 1.7/1000 patient days. 12/2110 (0.6%) infants had an early-onset sepsis. With regard to late-onset sepsis, 235 episodes occurred in 203/2110 (9.6%) VLBW infants. In table 1, the clinical characteristics of infants stratified to occurrence of late-onset sepsis are given. We noted a lower gestational age and birth weight in the late-onset sepsis group. Furthermore, infants with late-onset sepsis were more likely to be born by spontaneous vaginal delivery (16.3% vs. 7.9%) and emergency Caesarean section (15.3% vs. 8.5%) and had less frequent coverage with antenatal steroids (86.9 vs. 91.8%) than infants without late-onset sepsis (table 1).

**Table 1:** Clinical characteristics of the VLBW cohort stratified to occurrence of late-onset sepsis (LOS).

	No LOS	LOS patients	p#	total
**Number of infants**	1907	203		2110
**Gestational age** (mean/median, weeks)	28.9/28.9	26.9/26.7	<0.001	28.7/28.7
**Birth weight** (mean/median, grams)	1077/1100	878/810	<0.001	1058/1090
**Gender** (male, %)	49.8	50.7	0.8	49.9
**Multiples** (%)	32.8	28.6	0.22	32.4
**Inborn** (%)	97.5	97	0.68	97.5
**Mode of delivery,** vaginal (%)	7.9	16.3	<0.001	8.7
Elective C/section (%)	83.7	68.3	<0.001	82.2
Emergency C/section (%)	8.5	15.3	<0.001	9.1
**PPROM** (%)	29.6	28.5	0.75	29.5
**SGA** (%)	18.5	18.2	0.9	18.4
**Antenatal steroids** (%)	91.8	86.6	0.013	91.3
**Maternal descendence,** Germany (%)	73.2	63.7	0.059	72.3
Europe/Russia (%)	10	14.9		10.5
Middle East/Turkey (%)	10.8	14.9		11.2
Asia (%)	1.9	2		1.9
Africa (%)	2.6	2		2.5

**Legend:** PPROM Preterm premature rupture of membranes, C/section Caesarean section, SGA small-for-gestational-age (<10th Voigt percentile).

# p-values were derived from chi-square test if not otherwise indicated (* Mann-Whitney-U test).

### Pathogenic Spectrum of Late-onset Sepsis Episodes

In table 2 the pathogenic spectrum of blood-culture proven sepsis episodes is summarized. In 182/235 (77.4%) late-onset sepsis episodes gram-positive bacteria were documented, while coagulase negative staphylococci were found to be the most predominant pathogens (48.5%, 95%CI: 42.01–55.01). No Methicillin-resistant *Staphylococcus aureus* (MRSA) sepsis was documented. 10/235 (4.3%, 95%CI: 1.68% –6.83%) late-onset sepsis episodes were caused by *Candida spp*. and 43/235 (18.3%) episodes were caused by gram-negative bacilli. We noted that *Klebsiella spp*. played a major role as causative agent accounting for 22/43 gram-negative late-onset sepsis episodes followed by *Enterobacter spp.* (10/43) and *E. coli* (9/43). This prompted us to further investigate whether microclusters (linked cases with the same bacterium within three months) occur within the low-risk setting of the GNN cohort.

**Table 2:** Pathogenic spectrum of blood-culture proven sepsis episodes.

	Late-onset sepsis	Total
Pathogen	n; Estimate(95% CI)*	n; Estimate(95% CI)*
*Klebsiella species*	22; 9.36 (5.34–13.38)	22; 8.91 (4.48–13.33)
*Escherichia coli*	9; 3.83 (1.38–6.28)	14; 5.67 (2.48–8.86)
*Enterobacter cloacae*	10; 4.26 (1.69–6.82)	10; 4.05 (1.41–6.68)
*Enterococcus species*	15; 6.38 (3.26–9.51)	16; 6.48 (3.01–9.95)
*Staphylococcus aureus*	47; 20 (14.9–25.1)	47; 19.03 (12.23–25.83)
*CoNS*	114; 48.51 (42.01–55.01)	114; 46.15 (33–59.31)
*Group B streptococci*	5; 2.13 (0.28–3.97)	7; 2.83 (0.64–5.02)
*Other streptococci*	1; 0.43 (−0.41–1.26)	2; 0.81 (−0.33–1.95)
*Serratia*	1; 0.43 (−0.41–1.26)	1; 0.4 (−0.39–1.2)
*Pseudomonas aer.*	1; 0.43 (−0.41–1.26)	2; 0.81 (−0.33–1.95)
*Listeria monocytogenes*	1; 0 (0–0)	1; 0.4 (−0.39–1.2)
*Candida*	10; 4.26 (1.68–6.83)	11; 4.45 (1.66–7.24)
**Total**	**235**	**247**

* Estimate: estimated risk for specific pathogen/100 cases of blood-culture proven sepsis.

### Microclusters of Late-onset Sepsis

4 out of 23 centers had no case of sepsis with gram-negative bacilli or *Enterococcus spp.* in the study period. Of the remaining 19 centers, 7 hospitals (36.8%) were documented with a total of 11 microclusters. These microclusters were noted in 26 infants including 2 multiples (8 microclusters with 2 linked cases, 2 microclusters with 3 linked cases and 1 microcluster with 4 linked cases). Microclusters occurred in 16/22 cases caused by *Klebsiella spp.*, 4/9 cases caused by *E. coli*, 2/8 cases caused by *Enterobacter spp.* and 4/13 cases caused by *Enterococcus spp.*

Time intervals (median; 25^th^/75^th^ percentile in days) between occurrence of the first patient’s sepsis in the cluster and the linked cases were as follows: *Klebsiella spp.* (14.1; 1–27 days), *E.coli* (23.5; 8–39 days), *Enterobacter spp.* (26 days), all gram-negative bacilli (23.0, 5–27 days), *Enterococcus spp.* (56.5; 43–70).

### Outcome of Patients within a Cluster of Sepsis

In table 3, the clinical characteristics and outcomes of infants are described in relation to occurrence of sepsis within a cluster (n = 26 single sepsis cases, n = 11 first patients in a cluster, n = 15 linked cases in a cluster). No differences in outcomes were noted between these groups (table 3). When we restricted the analysis to gram-negative sepsis only, we noted by trend a higher rate of severe complications or death in the first patient of the cluster (7/9 infants; 77.8%) compared to their linked cases (6/13; 46.2%) or single cases (6/17; 35.3%).

**Table 3:** Outcome of patients in microclusters of blood-culture proven sepsis.

	Sporadic cases	Linked cases	First patient in cluster	total	p
**Number of infants**	26	15	11	52	
**Gestational age** (mean/median,weeks)	26.4/26.2	27.9/27.7	26.4/25.9	26.8/26.5	0.16*
**Birth weight** (mean/median, grams)	840/790	986/1140	810/755	876/790	0.11*
**IVH** (%), **total**	34.6	20	36.4	30.8	0.56
Grade 1	11.5	6.7	0	7.7	
Grade 2	15.4	0	18.2	11.5	
Grade 3	0	0	0	0	
Grade 4	7.7	13.3	18.2	11.5	
**Surgery PDA** (%)	4	0	18.2	5.9	0.13
**Surgery NEC/FIP** (%)	24	20	27.3	23.5	0.9
**Treatment ROP** (%)	8.3	0	10	6.1	0.49
**BPD** (%)	44.4	28.6	18.2	33.3	0.34
**Severe complication**	34.6	46.7	63.6	44.2	0.26
**or death** (%)					
**Death** (%)	7.7	20	30	15.7	0.26

**Legend:** IVH intraventricular haemorrhage; Surgery for PDA Patent Ductus arteriosus, NEC necrotizing enterocolitis, FIP focal intestinal perforation, ROP retinopathy of prematurity (kryo- or laser therapy); BPD bronchopulmonary dysplasia; severe complication: IVH grade IV, posthaemorrhagic hydrocephalus with need for VP shunt, periventricular leukomalacia, surgery for NEC/FIP or ROP and BPD;

p-values were derived from chi-square test if not otherwise indicated (* Mann-Whitney-U test comparing linked cases vs. first patient in cluster.

## Discussion

In the GNN study period 2009/2010 an overall risk for blood-culture proven sepsis of 11.4% was documented for all eligible infants. Infants enrolled in GNN centers with recruitment of ≥50 infants during the study period had an overall risk of 10.2% and late-onset sepsis risk of 9.6% with birth weight, gestational age and lack of antenatal steroid coverage being major risk factors. In comparison with previous data of our network (14.8 vs. 9.6% of late-onset sepsis) we were able to confirm that participation in a multicenter surveillance system reduces neonatal nosocomial bloodstream infections [10]. The pathogenic spectrum of late-onset sepsis episodes with predominance of gram-positive bacteria as causative agent in >70% and an 18% rate of gram-negative sepsis is comparable with the results of other infection surveillance networks [1]–[2], [18]–[19]. The NeonIN surveillance network in England documented sepsis episodes in all newborns not only restricted to VLBWs cared for in tertiary level units [13]. In the English cohort, the rate of gram-negative late-onset sepsis was higher (42%). While *Klebsiella* spp. played a major role in our cohort (22/43; 51.1% of gram-negative episodes), only 20% (37/177 cases) of gram-negative sepsis were associated with *Klebsiella spp*. in the NeonIN surveillance report.

So far, data on epidemic clusters of sepsis are limited to a few reports on outbreaks with common cross-transmitted pathogens. Modern molecular methods allow for the identification of a common source in at about one half of all outbreaks [15], [20]. In other examples, however, epidemiological surveys failed to identify the causes of the emergence of outbreak strains in NICU settings [9]. Therefore it is essential to collate data on the significance of epidemic microclusters of sepsis in surveillance networks to adapt clinical practice, inform policy and further improve quality of care. In order to do that, an adequate, internationally accepted definition of “cluster of sepsis” is needed. In this study, we decided to use the timeframe of 90 days to define timely linked sepsis cases in one center with the same bacterial spp. (microcluster). Using this definition the significant number of 11 epidemic microclusters of blood-culture proven sepsis in 7 NICUs (9/11 with gram-negative bacteria, 2/11 with *Enterococci*) is the main finding of this report. Even if we modified the definition with regard to timeframe of linkage to 30 days, 7/11 microclusters would have been detected, all of them involving gram-negative bacilli. In line with this, the second important experience in our cohort is that the time interval between occurrence of the first patient’s *Klebsiella spp.* sepsis in the cluster and the linked cases event was a median of 14.1 days (25^th^/75^th^ percentile: 1–27 days). These data suggest that there is a highly vulnerable time window of sepsis clustering after the first infant has been diagnosed even in a low-risk setting such as GNN. Therefore immediate reinforcement of strict adherence to hand hygiene policies, rigorous environmental hygiene, cohorting and isolation of other colonized infants as important measures in the proactive containment of an epidemic microcluster seems to be crucial [9], [21]. Further sepsis prevention must include evaluation of workload and adaptation of resources in the NICU on a regular basis, especially at a time when nursing staff is being reduced in stressed, high-throughput systems due to economical restrictions [11], [22].

We also evaluated the outcome data of sporadic sepsis patients in a NICU compared to sepsis patients in clusters and found no differences. When we restricted the analysis to gram-negative sepsis only, we noted a trend to a lower gestational age and a higher rate of severe complications or death in the first patient of the cluster compared to either their linked cases or independent sporadic sepsis cases. This observation merits further investigation in a larger epidemiological study. Our approach is limited by the fact that we are not able to provide data on the genotypic concordance of sepsis strains in one cluster as it has been demonstrated with pulsed-field gel electrophoresis in single or two-center investigations [9], [23]. The strength of our approach is the on-site monitoring and approval of clinical and microbiological data by a neonatologist which may be advantageous over computer/web-based surveillance systems without monitoring [24].

In summary, there is probably an underestimation of microclusters of gram-negative sepsis even in low-risk settings. The dramatic consequences of infections underline the need for additional research on the microbiomic constitution of the flora of VLBW and the mode of acquisition gram-negative bacteria. Future surveillance networks therefore require the combined effort of clinical and molecular epidemiology providing information of both, the pathogen (antimicrobial resistance patterns) and the host (whether colonized or infected) to elucidate its interaction in the cluster situation.
